# Detection of Myofascial Herniation on Dynamic Sonography and Magnetic Resonance Imaging

**DOI:** 10.1155/2016/4245189

**Published:** 2016-01-14

**Authors:** Sanjay M. Khaladkar, Sushen Kumar Kondapavuluri, Anubhav Kamal, Raghav Kalra, Vigyat Kamal

**Affiliations:** Department of Radio-Diagnosis, Dr. D. Y. Patil Medical College, Pimpri, Pune 411018, India

## Abstract

Muscle hernia is an uncommon cause of leg swelling. It can be detected in the early stages only if there is a high index of suspicion. It is common in lower extremity compared to the upper extremity. Tibialis anterior muscle in the leg is commonly involved. Dynamic sonography and magnetic resonance imaging (MRI) are the mainstay in their diagnosis, which demonstrate a facial defect with herniation of muscle fibers. We report a case of 23-year-old male patient who presented with a painless swelling in the anterolateral aspect of the left upper leg. Dynamic sonography done with high-resolution probe demonstrated a defect in fascia overlying tibialis anterior with herniation of outer muscle fibers which increased during dorsiflexion and reduced in the supine position at rest. MRI of the left leg confirmed the findings.

## 1. Introduction

Muscle hernia is also called myofascial herniation. Muscle herniation of the lower extremity is a rare entity and is often asymptomatic. Symptomatic muscle hernias commonly occur in the leg and cause chronic leg pain due to nerve involvement. The focal fascial defect with resultant muscle herniation into subcutaneous fat presents clinically as a soft tissue mass. Clinicians must remember to consider myofascial herniation in the differential diagnosis of chronic leg pain or neuropathy. We present a case of tibialis anterior muscle herniation due to overlying fascial defect presenting as a palpable mass detected on dynamic sonography and MRI.

## 2. Case Report

**Figure 1:**
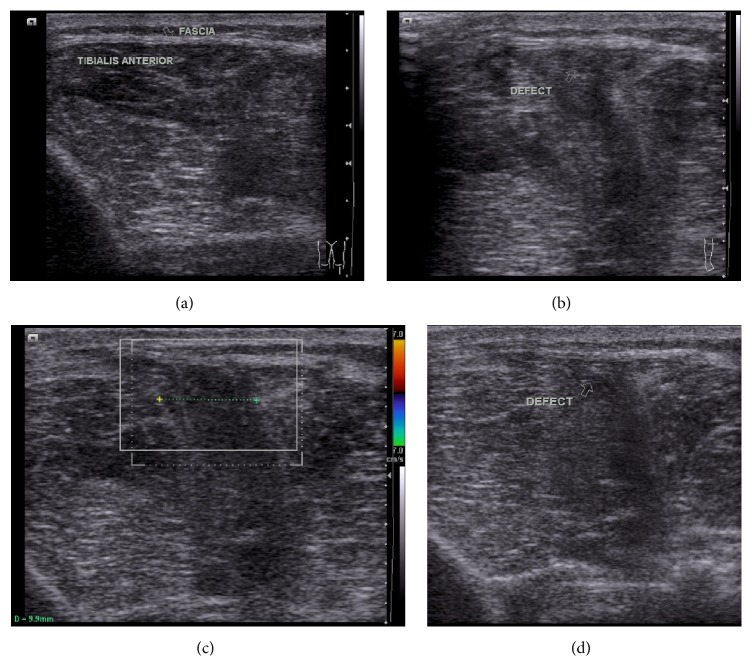
(a) Longitudinal section on the anterolateral aspect of left upper leg superior to the site of muscle hernia showing intact echogenic fascia with underlying tibialis anterior muscle. (b) Transverse section on the anterolateral aspect of left upper leg in standing position showing a defect in echogenic fascia (marked by arrow) with an increase in herniation of underlying tibialis anterior muscle through the defect. (c) Transverse section on the anterolateral aspect of left upper leg in standing position showing the defect of size 9.9 mm in echogenic fascia with herniation of underlying tibialis anterior muscle through the defect. (d) Longitudinal section on the anterolateral aspect of left upper leg at rest at the site of muscle hernia showing a defect in echogenic fascia with herniation of underlying tibialis anterior muscle through the defect (marked by arrow).

**Figure 2:**
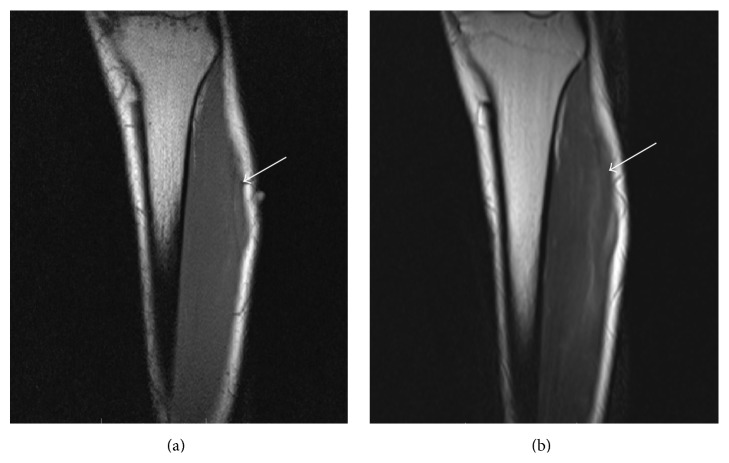
(a) Coronal section of left leg T1-weighted sequence showing defect at the level of the marker (marked by arrow) with outward bulging of underlying tibialis anterior through the defect. (b) Coronal section of left leg T2-weighted sequence outward bulging of tibialis anterior due to muscle herniation through the defect (marked by arrow).

**Figure 3:**
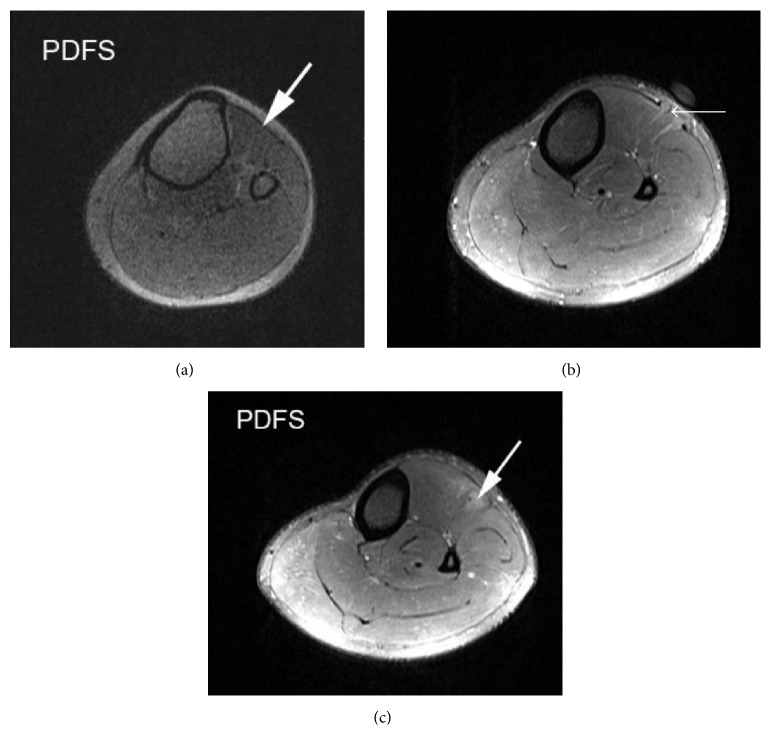
(a) Axial section of left upper leg PDFS sequence showing intact fascia overlying tibialis anterior muscle (marked by arrow). (b) Axial section of left upper leg PDFS sequence showing a defect in the fascia (marked by arrow) overlying tibialis anterior muscle at the level of the marker with resultant herniation of underlying muscle fibers. (c) Axial section of left upper leg PDFS sequence inferior to the level of defect showing intact fascia with a diffuse hyperintense signal in underlying tibialis anterior muscle due to muscle edema (marked by arrow).

A 23-year-old male presented with painless swelling in the anterolateral aspect of the left upper leg for 1-year duration. The swelling was prominent in standing position and reduced in lying down position at rest. There was no history of trauma. On local examination, a diffuse swelling appeared in the anterolateral aspect of the left upper leg in standing position which accentuated dorsiflexion of the foot and reduced in the supine position. It was nontender. Overlying skin temperature and color were normal. Local ultrasonography (USG) done with a high-resolution linear probe (frequency: 7 to 12 MHz) in standing position showed intact echogenic fascia overlying tibialis anterior superior to site of swelling (Figure 1(a)) and a defect of size 9.9 mm in echogenic fascia in the anteroposterior direction overlying the tibialis anterior in upper third of leg, extending superoinferiorly over a length of approximately 5.5 cm at site of swelling. There was herniation of the outer fibers of tibialis anterior muscle through this defect (Figures 1(b) and 1(c)). Muscle hernia showed reduction in lying down position (Figure 1(d)). Limited MRI (static) of the left leg was performed at rest for confirmation of USG findings by taking coronal T1 and T2 sequences (Figures 2(a) and 2(b)) followed by axial PD fat saturated sequence (Figures 3(a) and 3(b)) after placing a marker at the site of diffuse swelling. MRI confirmed a fascial defect overlying tibialis anterior measuring 1.2 cm in width extending superoinferiorly over a length of 5.5 cm with resultant muscle herniation and outward bulge of tibialis anterior. Outer fibers of tibialis anterior revealed subtle hyperintense signal on PD fat saturated sequence suggestive of muscle edema (Figure 3(c)). The patient was referred to the orthopedic department for repair of myofascial defect which he refused.

## 3. Discussion

Ihde provided original investigation and ground work on lower extremity muscle hernias [1]. Largest contribution is due to the combined efforts of military surgeons who observed muscle hernias in actively training military recruits; otherwise it occurs as sporadic cases. The majority of the earlier work of leg hernias is found in French medical literature which is based on military experience [1, 2]. Ihde in 1929 reported a series of 12 patients with hernias of leg and acknowledged the earlier description by Richet in 1853 and first actual definition provided in 1861 by Mourlon as “the displacement of muscle beyond its ruptured aponeurosis” [2]. Muscle hernias are caused by a focal defect in the fascial sheath. It is classified by Ihde [1] as constitutional (congenital) and traumatic (acquired). Congenital causes may be due to general weakness in muscular fascia (mesodermal insufficiency) or occur at sites of perforating nerves and vessels. Acquired cases are secondary to trauma as seen in penetrating trauma, direct trauma causing close fracture with fascial tear, and indirect trauma (force applied to contracted muscle causing fascial rupture) [3]. Increase in intracompartmental pressure seen in muscle hypertrophy or chronic exertional compartment syndrome (CECS) potentiates herniation [4]. Regular cardiovascular exercise and physical activity cause muscle hypertrophy with a 20% increase in muscle volume. CECS is a reversible form of abnormally increased intramuscular pressure during physical exertion or exercise secondary to noncompliance of osteofascial tissue to exercise-induced increase in muscle volume [4]. The true incidence of a leg hernia is not known, as most are asymptomatic and remain undiagnosed and are not brought to the attention of the physician. About 200 cases of muscle hernias are described in the literature [5]. Military soldier, athletes, mountain climbers, and skiers are the demographic population at highest risk who are prone to CECS. Fascial defects are found in 15–50% of patients undergoing surgery, even with normal preoperative examination [6].

The anterolateral tibial compartment is the commonest site due to superficial and tight fascial compartment [5]. A high index of suspicion and awareness of a muscle hernia helps in its early diagnosis. In the leg, tibialis anterior is the most commonly involved muscle and most reported in the literature. The fascia of tibialis anterior is more vulnerable to trauma as it is the weakest fascial point in the lower extremity [3]. Other muscles involved are peroneus longus, peroneus brevis, extensor digitorum longus, gastrocnemius, and flexor digitorum longus. Multiple hernias within the same muscle and bilateral symmetrical involvement may also be seen in the thigh. Iatrogenically induced hernia, involving rectus femoris and vastus lateralis, can occur as a complication following an anterolateral thigh perforator flap and after fascia lata harvest for cruciate ligament repair. The lower incidence of a muscle hernia in a peroneal compartment is due to a relatively large subcutaneous surface area aligning for increase compliance.

On clinical examination, muscle hernia may present as a palpable bulge, soft tissue mass, or subcutaneous nodule. It may be solitary, multiple, or bilateral. It is usually reducible on lying down. It may be irreducible when there is strangulated muscle. The patient may complain of pain, discomfort, weakness, cramping, or neuropathy. It worsens with standing and physical activity. On examination, local tenderness and decreased sensation may occur if there is associated nerve involvement. Herniation of tibialis anterior is pronounced with resisting dorsiflexion of the foot. If reducible, the fascial defect may be appreciated. A muscle hernia is commonly seen in adolescence and young adults who present with swelling which appears or enlarges on standing erect or when affected muscle is contracted. The swelling reduces when the patient is supine or muscle is relaxed. Differential diagnosis of muscle hernias are varicosities, angiomas, arteriovenous malformation, lipomas, ruptured muscle (a pseudohernia), and soft tissue tumors [3]. Dynamic sonography and MRI are diagnostic in detecting a myofascial defect. Dynamic sonography detects muscle bulge through the fascial defect on muscle contraction and reduces on relaxation. Sonography is advantageous as it is real time, muscle herniation is detected during dynamic examination, and nature of the lesion can be shown to the patient which helps in reassurance. The examination is done in standing position or by contracting the muscle. Marking of overlying skin is done as the mass may be difficult to be felt. High-frequency probe (more than 7.5 MHz) is used after liberal application of coupling gel. Gain and focus are set to optimize near field. The transducer is applied lightly as heavy pressure can efface a hernia [7].

Normal muscle is covered by thin echogenic fascia. In mild cases, fascia is thinned out with no demonstrable defect. There is a mild muscle bulge with an elevation of the overlying fascia. In overt cases, the margins of the defect are clearly defined with outward bulge and herniation of muscle through the defect. The herniated muscle and adjoining nonherniated muscle are less echogenic than normal muscle due to anisotropy or atrophy caused by low-grade repetitive trauma. Frank's hernia gives mushroom-like appearance causing convex superficial contour with herniated muscle overlapping the fascial defect. Spoke-like appearance caused by normal echogenic fibroadipose septae is seen as they are pinched through the fascial defect as they radiate from the center of the defect in the fascia. In a few cases, a prominent arterial pulsation can be identified on contour or power Doppler supporting the theory of muscle herniation at sites of weakness where vessels penetrate the fascia. USG is relatively easy and of low cost. 3D dynamic USG with surface rendering improves visualization of focal planes and the muscular protrusion and is superior to traditional 2-dimensional ultrasonography [7].

Though expensive, MRI confirms muscle herniation in equivocal USG findings. It better visualizes musculofascial demarcation and allows quantification of fascial splitting and muscle herniation. Dynamic ultrasonography and MRI incorporate fast imaging with forced muscular movements (dorsiflexion and plantar flexion at the ankle) and enable better visualization and pinpointing of the hernia and fascial defect. The defect is in the deep layer of the deep fascia. It may thin or elevate the superficial layer of deep fascia which is overlying the muscle [8–10]. Treatment of tibialis anterior herniation is controversial. Asymptomatic hernias require no specific treatment and rupture reassurance. Mildly symptomatic hernias often respond to conservative treatment with restriction of exercise and use of elastic support. The surgical option is the direct closure of the defect. However, it causes an increase in intracompartmental pressure accentuated in the postoperative period by edema causing recurrence. The fascial opening can be repaired with a patch of fascia lata or periosteal graft. However, this may be a cause of compartment syndrome and need an additional donor. Hence, recommended treatment is longitudinal fasciotomy which prevents the development of compartment syndrome [11].

## 4. Conclusion

A muscle hernia is an uncommon cause of swelling in the lower extremity which may cause chronic leg pain and neuropathy. It is common in lower extremity compared to the upper extremity. Tibialis anterior is commonly involved. Dynamic sonography and MRI are the investigations of choice in their detection.

## Abbreviations

MRI: Magnetic resonance imaging
CECS: Chronic exertional compartment syndrome
USG: Ultrasonography.
